# Anti‐PD‐1 antibodies in metastatic uveal melanoma: a treatment option?

**DOI:** 10.1002/cam4.887

**Published:** 2017-06-21

**Authors:** Carolin Bender, Alexander Enk, Ralf Gutzmer, Jessica C. Hassel

**Affiliations:** ^1^ Department of Dermatology and National Center for Tumor Diseases University Hospital Heidelberg Im Neuenheimer Feld 440/460 Heidelberg 69120 Germany; ^2^ Department of Dermatology and Allergy Skin Cancer Center Hannover Hannover Medical School Ricklinger Str. 5 Hannover 30449 Germany

**Keywords:** Immunotherapy, intraocular melanoma, nivolumab, PD‐1 Ab, pembrolizumab, Uveal melanoma

## Abstract

Uveal melanomas (UMs) are a rare form of cancer with clinical and pathological characteristics distinct from cutaneous melanomas. Ipilimumab has shown efficacy and safety in the treatment of metastatic UM. This provides a rationale for treatment with other immune checkpoint inhibitors. This is a retrospective review of 15 patients with metastatic UM treated between June 2014 and February 2016, who received treatment with the anti‐PD‐1 Abs pembrolizumab or nivolumab. Patients were treated at two German university hospitals. Therapy was administered at the approved dosing schedules of 2 mg/kg q3w for pembrolizumab and 3 mg/kg q2w for nivolumab. Treatment was given until first tumor assessment and continued if tumor assessment showed disease control. Tumor assessments were performed at baseline and following scans every 12 weeks. Patients were monitored throughout for adverse events. Best response to treatment was stable disease in four patients. Eight out of 15 (53%) patients received treatment until first tumor assessment. As of February 2016, median progression‐free survival (PFS) is 3 months (range 0.75–6.75 months) and overall survival (OS) is 5 months (range 1–16 months). Eight out of 15 (53%) patients are still alive (two patients lost to follow‐up) with one out of four patients is in ongoing disease control. Patients with multiple organ metastases and elevated serum lactate dehydrogenase did not respond well to treatment. No objective response to PD‐1 Ab therapy was seen. Best response to treatment was stable disease in four patients. Treatment was well tolerated with manageable toxicity.

## Introduction

Uveal melanomas (UMs) are a rare form of cancer with clinical and pathologic characteristics distinct form cutaneous melanomas (CMs). Being the most common primary intraocular tumor the UM involves the vascular layers of the eye. Uveal melanomas account for fewer than 5% of melanomas and carry a poor prognosis with half of the patients developing metastatic disease despite enucleation and/or radiotherapy of the primary lesion 1. Unlike cutaneous melanomas, about 80% of UMs show mutations in G‐protein *α*‐subunits q (GNAQ) and 11 (GNA11) 2.

Uveal melanomas predominantly metastasize to the liver which can be the sole site of metastasis 2. Median survival time for patients with metastatic disease is approximately 12 months as response rates to therapy are poor and as there are limited treatment options available. Survival rates have not improved in the last 20 years 1.

### Rationale

In metastatic UM, ipilimumab has shown efficacy and safety in previous reports 3, 4. In a case series, two out of 56 (3.6%) patients experienced partial response (PR) while 12 patients (21.4%) showed disease stabilization 1. Among another 82 UM patients treated through an expanded access program (EAP) in Italy, four (5%) had immune‐related (ir) PR and 24 (29%) had ir stable disease (SD) lasting for ≥3 months for a disease control rate of 34% 4.

In two prospective clinical trials, ipilimumab showed limited clinical activity in patients with metastatic UM. In the phase II DeCOG‐study, patients received up to four cycles of ipilimumab administered at a dose of 3 mg/kg q3w. Median overall survival (OS) was 6.8 months (95% CI: 3.7–8.1), and median progression‐free survival (PFS) was 2.8 months (95% CI: 2.5–2.9). Sixteen patients had stable disease (47%), none experienced partial or complete response. One‐year and two‐year OS rates were 22% and 7%, respectively 5. An interim analysis of the GEM‐1 trial showed preliminary data from 31 patients. With a median follow‐up of 5.5 (CI 95%: 3.4–11.1) months, 13 patients were evaluated for response: one patient experienced PR (7.7%) and six patients experienced SD (46.2%). Ipilimumab was administered at doses of 10 mg/kg IV q3w for four doses (induction) followed by q12w (maintenance) until progression, intolerance, or withdrawal 6.

As PD‐L1 expression is found in UM cells, further investigation of treatment strategies targeting PD‐1/PD‐L1 is reasonable 7.

## Materials and Methods

### Patients

Data from patients with metastatic UM treated with pembrolizumab or nivolumab at two German university hospitals were retrospectively analyzed.

The review comprised five patients who were enrolled in an EAP (NCT02083484). All other patients received anti‐PD‐1 Ab treatment after European Medicines Agency (EMA) regulatory approval.

In the EAP, eligible patients ≥ 12 years of age with unresectable stage III or IV cutaneous, metastatic ocular, or mucosal melanoma who had progressed on prior therapy (ipilimumab and targeted therapy when indicated) were treated with pembrolizumab. An Eastern Cooperative Oncology Group (ECOG) performance status of 0–1 8 was required for inclusion as well as recovery to grade 0–1 (according to NCI CTCAE v4.0 9) from AEs due to prior therapy. Patients with asymptomatic, pretreated brain metastases at baseline were eligible.

Major exclusion criteria were previous treatment with a PD‐1 or PD‐L1 blocking agent, current systemic immunosuppressive therapy, and active infection or active autoimmune disease.

### Study design and assessments

Therapy was administered at the approved dosing schedules of 2 mg/kg q3w for pembrolizumab and 3 mg/kg q2w for nivolumab and continued if tumor assessment showed response to therapy according to immune‐related response criteria (irRC) 10. Response outcome was described after the assessment scan at 12 weeks compared with baseline. In the EAP, dose reduction or modification was not allowed, but dose omission or discontinuation was recommended when necessary.

All patients were monitored throughout for AEs, including irAEs. AEs were managed using protocol‐specific guidelines and graded according to NCI CTCAE v4.0.

### Histology

Histological and chromogenic immunohistochemical stainings were performed on paraffin‐embedded tumor tissue taken before start of treatment of selected patients.

All immunostaining for CD3, CD4, CD8, CD20, and PD‐L1 was performed on adjacent sections to allow comparison of regional distributions of tumor‐infiltrating lymphocyte (TIL) subset infiltrates.

## Results

### Patients

Fifteen patients (six male, nine female) with UM received anti‐PD‐1 Abs at Hannover Medical School and Heidelberg University Clinic; baseline demographic and clinical characteristics are given in Table 1. Four patients were treated with nivolumab and 11 patients with pembrolizumab. Five out of 11 patients were treated inside the pembrolizumab EAP.

**Table 1:** Patients' baseline demographic and clinical characteristics

ID	Gender	Age	Date of first diagnosis	Mutational status	Sites of metastasis^a^	Systemic therapies before Anti‐PD‐1 Ab	Anti‐PD‐1 Ab^b^	Elevated serum LDH	No of treatment cycles	Best Response	Reason for discontinuation
1	Female	72	Feb 12	GNAQ & GNA11 wt, BRAF wt	1; 2; 4	Ipilimumab (1 cycle)	P‐EAP	yes	4	SD	PD
2	Female	79	1973	GNA11 mutation, cKIT wt	1; 3; 5; 6	Ipilimumab (4 cycles), chemotherapy, other therapies	P‐EAP	yes	3	not assessable	deterioration of health
3	Female	57	2002	GNAQ & GNA11 wt	1	Ipilimumab (1 cycle), chemotherapy	P‐EAP	yes	6	SD	PD
4	Female	54	Jan 13	GNA11 mutation	1; 2; 5; 6	targeted therapy, chemotherapy, ipilimumab (2 cycles)	P‐EAP	yes	2	not assessable	AE
5	Male	66	Nov 15	BRAF wt, GNA11/GNAQ not assessed	1	Ipilimumab (2 cycles)	P	yes	4	PD	PD
6	Female	64	Jan 02	GNA11 mutation, BRAF wt, cKIT wt,	1; 2	Ipilimumab (2 cycles), targeted therapy	P	yes	2	not assessable	deterioration of health
7	Male	38	Mar 14	GNAQ mutation, BRAF wt, cKIT wt	1	none	N	no	8	SD	flare of pre‐existing autoimmune disease
8	Female	79	Jan 14	triple wt, GNAQ & GNA11 wt	1; 2; 6	targeted therapy in clinical trial, Ipilimumab (4 cycles)	P	yes	1	not assessable	deterioration of health
9	male	58	Jan 13	GNAQ mutation, BRAF wt	1; 2; 6	none	P	no	4	PD	PD
10	Male	69	Jan 14	BRAF wt	1	none	N	no	4	PD	PD
11	Female	32	Jul 13	BRAF wt	6	none	N	no	6	pending tumor assessment	ongoing
12	Male	63	Feb 14	not assessed	2; 6	none	P	yes	6	SD	ongoing
13	Male	74	Jun 05	BRAF wt	1	none	N	yes	6	PD	PD
14	Female	62	Jun 09	not assessed	1	Ipilimumab (4 cycles)	P‐EAP/P	no	9	PD	PD
15	Female	64	Nov 11	not assessed	1; 2; 3; 6	chemotherapy	P	yes	3	PD	PD

SD, stable disease.

a 1 =  liver, 2 =  lungs, 3 =  brain, 4 =  adrenal gland, 5 =  lymph nodes, 6 =  other sites.

b N, Nivolumab; P, Pembrolizumab; P‐EAP, treatment inside pembrolizumab EAP.

All patients in the EAP had progressed on ipilimumab treatment and three of these had received additional systemic therapies including multiagent chemotherapy (2/3) and targeted therapy (1/3). Nivolumab was administered first‐line in all patients. Six out of 15 patients received anti‐PD‐1 treatment first‐line.

### Safety and tolerability

Eight out of 15 (53%) patients received treatment until first tumor assessment with one patient still being under treatment without first tumor assessment. Most common reason for early discontinuation was deterioration of general health. One case of suspected autoimmune hepatitis grade 1 occurred and one patient had to stop treatment because of a flare of his preexisting autoimmune disease.

### Clinical response

As of February 2016, median PFS is 3 months (range 0.75– 6.75 months) and OS is 5 months (range 1–16 months). Eight out of 15 (53%) patients are still alive (two patients lost to follow‐up). Concerning efficacy best response to treatment was SD in four patients. One out of four patients with initial disease control is in ongoing response. In the majority of patients, disease control was not durable and usually PD was seen before or at week 24 with progressive hepatic metastases.

Seven out of 10 (70%) patients with elevated serum lactate dehydrogenase (LDH) levels progressed on anti‐PD‐1 Ab treatment. Single organ metastasis was observed in two out of seven patients with SD. Two out of six patients in first‐line therapy achieved disease control with one patient still being in the first 12 weeks of treatment.

### Histology

We took a retrospective look at tissue samples taken before the start of anti‐PD‐1 treatment from patients achieving disease control. Tissue from patients 3 and 7 was available for staining.

**Figure 1:**
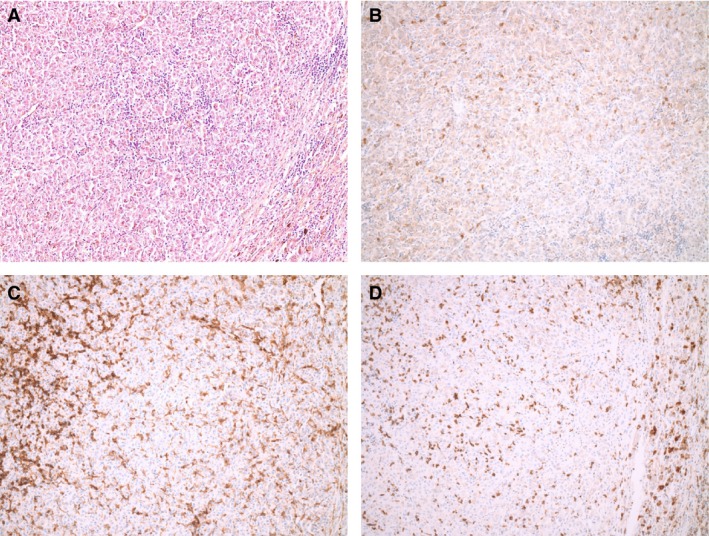
Pictures of PD‐L1, CD4, and CD8 expression using immunohistochemistry on tumor specimen of patient 3. (A) HE staining, (B) PD‐L1 expression, (C) CD4 expression, and (D) CD8 expression (all at magnification×100).

Tumor tissue of patient 3 showed a pronounced immune infiltrate in HE staining (see Fig. 1A). Immuno–histochemical staining for CD4 and CD8 expression revealed a high count of TILs (see Fig. 1C and D). Membranous as well as cytoplasmatic expression of PD‐L1 was observed in some parts the tumor tissue (see Fig. 1B).

**Figure 2:**
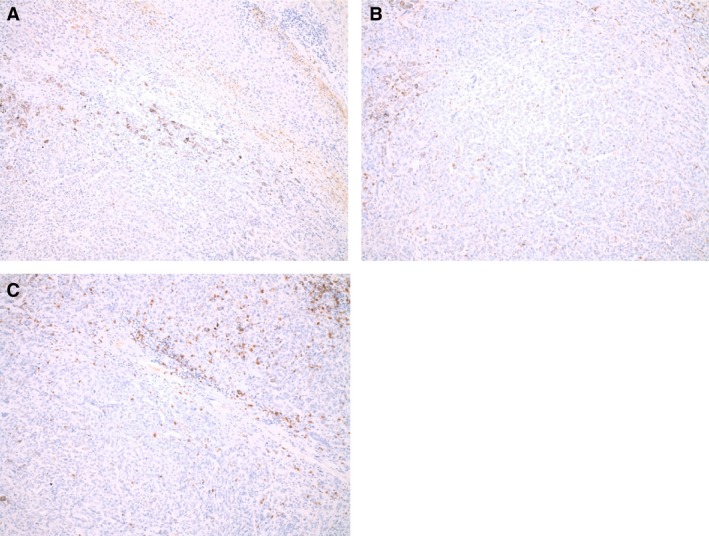
Pictures of PD‐L1, CD4, and CD8 expression using immunohistochemistry on tumor specimen of patient 7. (A) PD‐L1 expression, (B) CD4 expression, and (C) CD8 expression (all at magnification×100).

Staining of tumor tissue of patient 7 showed low expression of PD‐L1 (see Fig. 2A) and low counts of CD4‐ and CD8‐positive cells with high CD8/CD4 ratio (see Fig. 2B and C).

## Discussion

First reports of UM patients treated with PD‐1 Abs indicated good efficacy and safety for PD‐1 Ab treatment. Preliminary results of seven patients with metastatic UM receiving pembrolizumab inside a clinical trial showed one patient with irCR, one patient with irPR, and one patient with irSD 11.

In our patient group, response rates to anti‐PD‐1 Abs were low with limited clinical activity. Best response to anti‐PD‐1 Ab treatment was SD in four patients. One patient is still receiving treatment without first tumor assessment.

Thus, our results differ from the case series by Kottschade et al., who reported three out of seven patients (42.9%) achieving immune‐related disease control. Recently, Tsai et al 12. retrospectively analyzed a series of metastatic UM patients treated with pembrolizumab or nivolumab or PD‐L1 Ab atezolizumab. Overall response rate (ORR) was 3% and SD greater than 6 months was observed in four (7%) pts. Median PFS was 2.7 months (95% CI: 2.4–3.3) and median OS was 9.5 months (95% CI: 5.5–15), thereby reflecting our own experiences with PD‐1 Abs in UM.

Normal serum LDH, no previous ipilimumab, and presence of lung metastasis are correlated with better response to pembrolizumab in advanced CM and UM, whereas the presence of liver metastasis correlated with lower response to pembrolizumab. These correlations were observed regardless of BRAF status, presence of brain metastasis, or site of primary melanoma (CM vs. UM) 13.

UMs predominantly metastasize to the liver and all but two patients in this study had liver metastasis. In our group of patients, two out of six patients with sole hepatic metastasis responded to treatment probably due to lower tumor burden. Elevated serum LDH at the start of treatment was again shown to be a negative predictor for response despite of our small group of patients.

Treatment was well tolerated displaying the usual safety profile of anti‐PD‐1 Abs. One case of suspected autoimmune hepatitis grade 1 occurred in our patients.

PD‐L1 expression and high TIL grade in tumor samples have been associated with a favorable outcome in patients receiving immunotherapy 14. Yet, TIL infiltration in UM seems to be associated with a poor prognosis in contrast to epithelial carcinomas 15. Histological examination of tumor tissue of two patients achieving disease control showed PD‐L1 positive tumor samples. TIL grades differed greatly between the two specimens.

As there seems to be limited durable response in UM, anti‐PD‐1 Ab treatment could be part of a multimodal treatment approach combined with locoregional interventions. Currently, the study NCT01730157 looks at the efficacy of combined CTLA‐4 therapy and radioembolization in UM. Also, combined CTLA‐4/PD‐1 inhibition and treatment inside clinical trials should be considered in patients with multiple organ involvement as anti‐PD‐1 Ab monotherapy did not lead to high response rates in these patients. At present, two phase II trials are in progress for combination therapy of ipilimumab and nivolumab in UM.

In conclusion, this analysis is limited due to its retrospective nature and short follow‐up. Yet, real world data outside of clinical trials is needed as case numbers are low and evidence‐based guidelines for staging and treatment are missing.

## Conflict of Interest

Author C. Bender has received travel funds and a speaker honorarium from Merck Sharp and Dohme. Author A. Enk has received a speaker honorarium from BMS and Roche Pharma. He consults Biotest AG, MSD Oncology, Galderma Lab, Janssen, and AbbVie. Author R. Gutzmer has been paid honoraria by Roche, Bristol‐Myers Squibb, GlaxoSmithKline, Novartis, Merck Sharp and Dohme, Merck Serono, Almirall, Amgen, and Boehringer Ingelheim. He consults Roche, Bristol‐Myers Squibb, GlaxoSmithKline, Novartis, Merck Sharp and Dohme, LEO, Amgen, and Pfizer, and has received research funding from Roche, Novartis, Pfizer, and Johnson & Johnson. Travel expenses were paid by Bristol‐Myers Squibb and Roche. J.C. Hassel reports personal fees from Bristol‐Myers Squibb, GlaxoSmithKline, Merck Sharp and Dohme, Roche, Amgen, and Merck Serono.
